# Case report: Abrikossoff's tumor of the facial skin

**DOI:** 10.3389/fmed.2023.1149735

**Published:** 2023-05-31

**Authors:** Valeriu Ardeleanu, Radu Cristian Jecan, Marius Moroianu, Razvan Nicolae Teodoreanu, Tiberiu Tebeica, Lavinia Alexandra Moroianu, Florin Ciprian Bujoreanu, Lawrence Chukwudi Nwabudike, Alin Laurentiu Tatu

**Affiliations:** ^1^Faculty of Medicine, Doctoral School, “Ovidius” University, Constanţa, Romania; ^2^General Hospital “Căi Ferate, ” Galaţi, Romania; ^3^Arestetic Clinic, Galaţi, Romania; ^4^Faculty of Kinetotherapy, University “Dunărea de Jos, ” Galaţi, Romania; ^5^Department of Plastic Surgery and Reconstructive Microsurgery, Carol Davila University of Medicine and Pharmacy, Bucharest, Romania; ^6^Clinical Department of Plastic Surgery and Reconstructive Microsurgery, “Prof. Dr. Agrippa Ionescu” Emergency Clinical Hospital, Bucharest, Romania; ^7^Department of Dental Medicine, Faculty of Medicine and Pharmacy, “Dunărea de Jos” University, Galaţi, Romania; ^8^Medical Assistance Service of the Municipality of Galaţi, Galaţi, Romania; ^9^Leventer Medical Center, Bucharest, Romania; ^10^“Elisabeta Doamna” Psychiatry Hospital, Galaţi, Romania; ^11^Clinical Medical Department, Faculty of Medicine and Pharmacy, “Dunărea de Jos” University, Galaţi, Romania; ^12^Dermatology Department, “Sfanta Cuvioasa Parascheva” Clinical Hospital of Infectious Diseases, Galaţi, Romania; ^13^N. Paulescu National Institute of Diabetes, Bucharest, Romania; ^14^Multidisciplinary Integrated Center of Dermatological Interface Research MIC-DIR (Centrul Integrat Multidisciplinar de Cercetare de Interfata Dermatologica - CIM-CID), “Dunărea de Jos” University, Galaţi, Romania

**Keywords:** tumor, skin cancer, Abrikossoff, granular cell tumor, facial skin, Schwann cells, oral tumors

## Abstract

Abrikossoff tumors, also known as granular cell tumors (GCT), originate from Schwann cells. The most common location is in the oral cavity, followed by the skin, but they can also be found in the breast, digestive tract, tracheobronchial tree, or central nervous system. They can affect both sexes at any age, with a higher incidence between 30 and 50 years and a slight predisposition for female sex. They are usually solitary tumors but may also be multifocal. Most of the time, they are benign, with malignancy being exceptional in <2% of cases. Clinically, they appear as solid, well-defined, painless tumors, located subcutaneously with dimensions that can reach up to 10 cm. The definitive diagnosis is based on the immunohistochemical examination, and the treatment for benign tumors consists of surgical excision. Chemotherapy or radiotherapy may be required for malignant lesions, but the treatment regimens and their benefits remain unclear. This manuscript presents the case of a 12-year-old girl with a benign GCT, located in the skin on the mandibular line.

## 1. Introduction

Abrikossoff tumors, also known as granular cell tumors, are rare and often benign soft tissue tumors of Schwann cell origin (1). Although a granular cell tumor (GCT) usually develops in the skin or oral mucosa, it has been described as seen in many other organs (2). GCT typically presents as a solitary tumor, although it may sometimes be multiple, and in recent years, there have been reports of cases associated with Noonan syndrome and neurofibromatosis (3). It has also been described in association with other diseases. The vast majority of cases are reported in the skin and subcutaneous tissue. However, 2% of Abrikossoff tumors can be malignant (4). There is no predisposition for a certain sex although they can be found at all ages, and they predominate between the fourth and sixth decades of life (1, 5).

## 2. Case report

**Figure 1:**
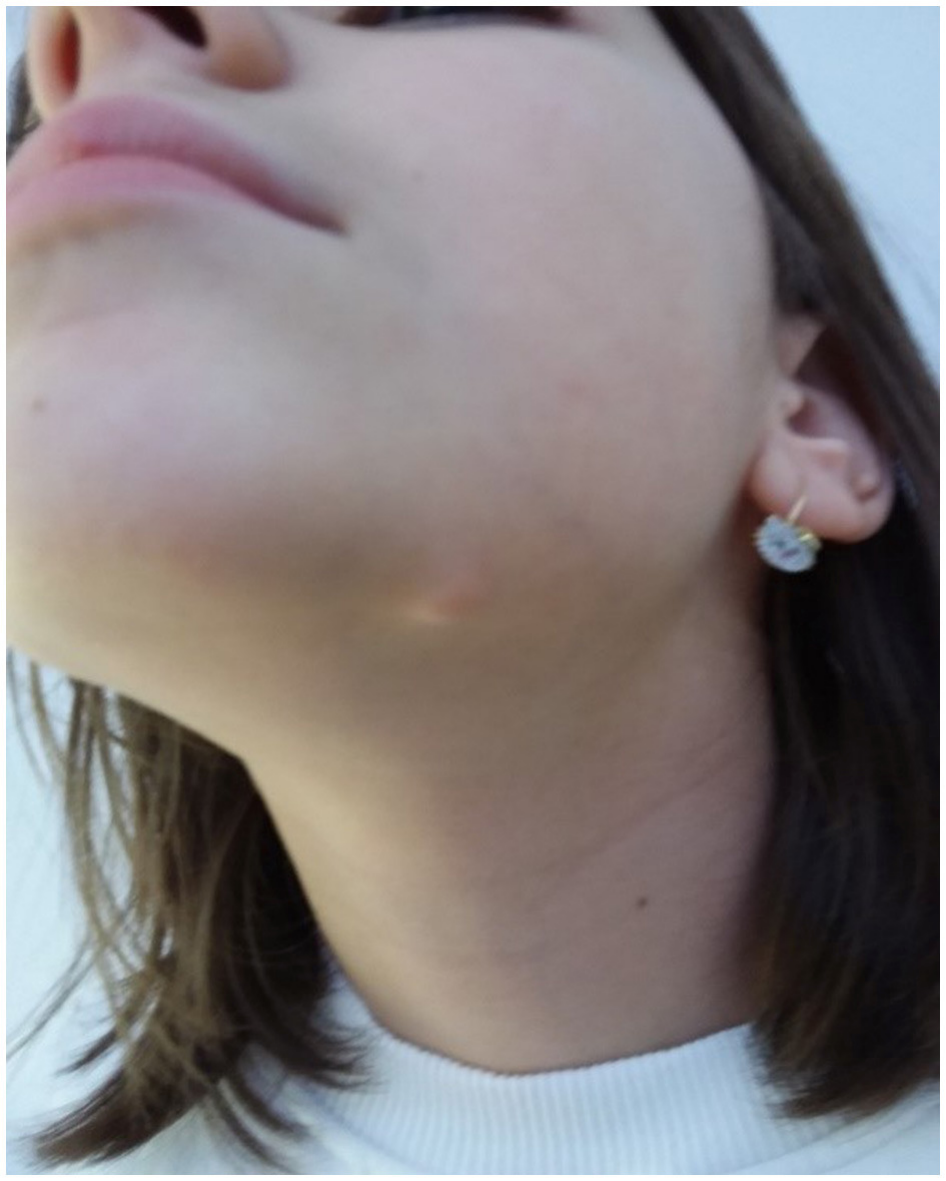
Well-defined, skin-colored, nodular lesion beneath the left mandible.

A 12-year-old female patient presented with a 12 mm mass in the area of the left mandible. Examination showed a 15 mm round, well-defined, non-tender, skin-colored nodular lesion, with a slightly depressed center (Figure 1).

**Figure 2:**
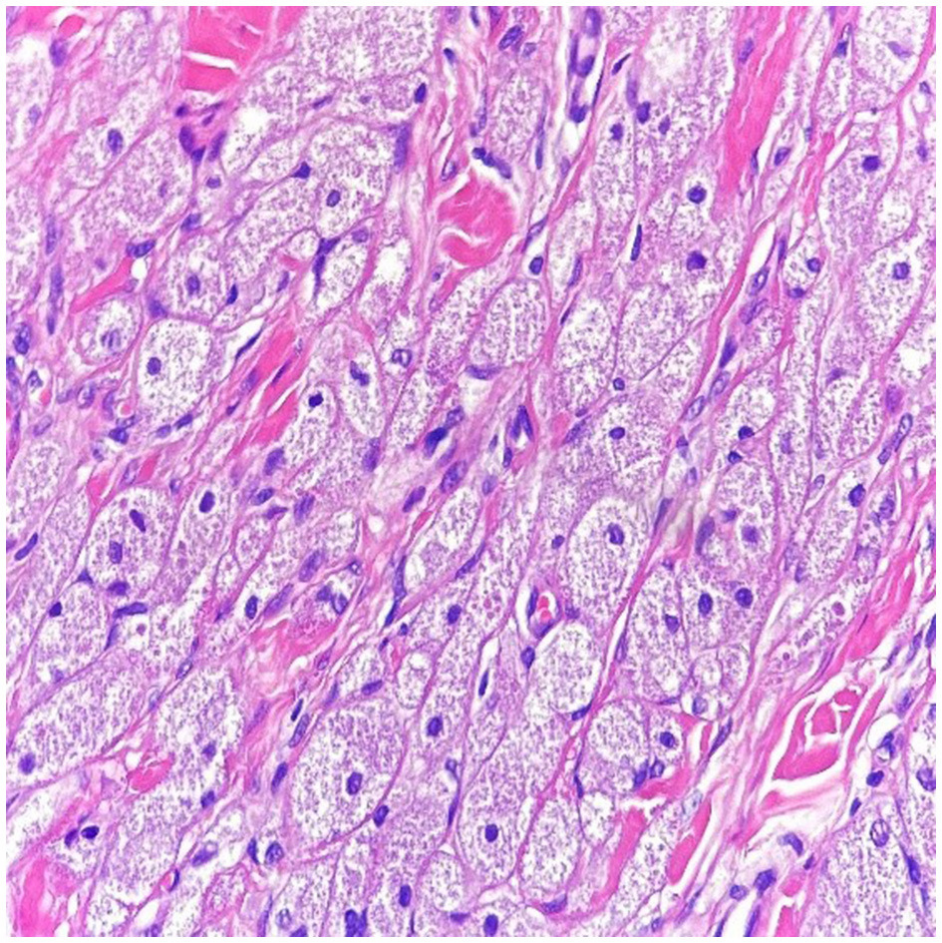
In high power magnification (10 x magnification-H & E stain), the cells have abundant eosinophilic granular cytoplasm with a small nucleus within a collagenous stroma.

**Figure 3:**
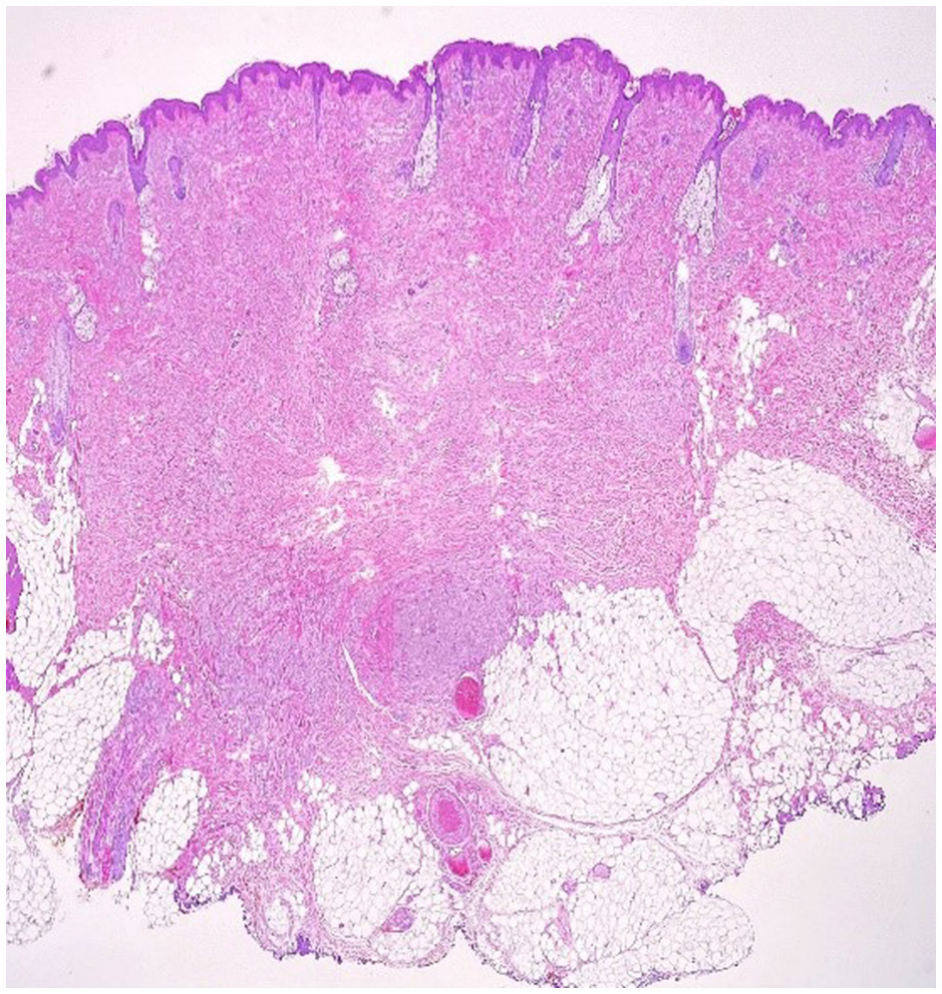
Cutaneous fragment with central fibrosis in the dermis, aside from this fibrous tissue, there is a non-encapsulated tumor proliferation with irregular borders and perineural extension (10 × magnification–H & E stain).

An excision biopsy under local anesthesia was carried out and atypical-appearing, partially fatty tissue was removed, which was sent for histopathology. An Abrikossoff tumor was diagnosed, and a wider excision was carried out. Immunohistochemistry of the first piece of the tissue showed a tumor containing residual granular Abrikossoff cells surrounding the dermal fibrous scar tissue. The tumor appeared to have been completely excised, with the exception of a tumor fascicle extending perineurally, involving the deep margins of the resected area. Immediately adjacent to and beneath, this is an area of tumor proliferation comprised of cubes, sheets, and arches of large cells with small, round nuclei, central nuclei with an abundant eosinophillic, granular cytoplasm (Figures 2, 3).

**Figure 4:**
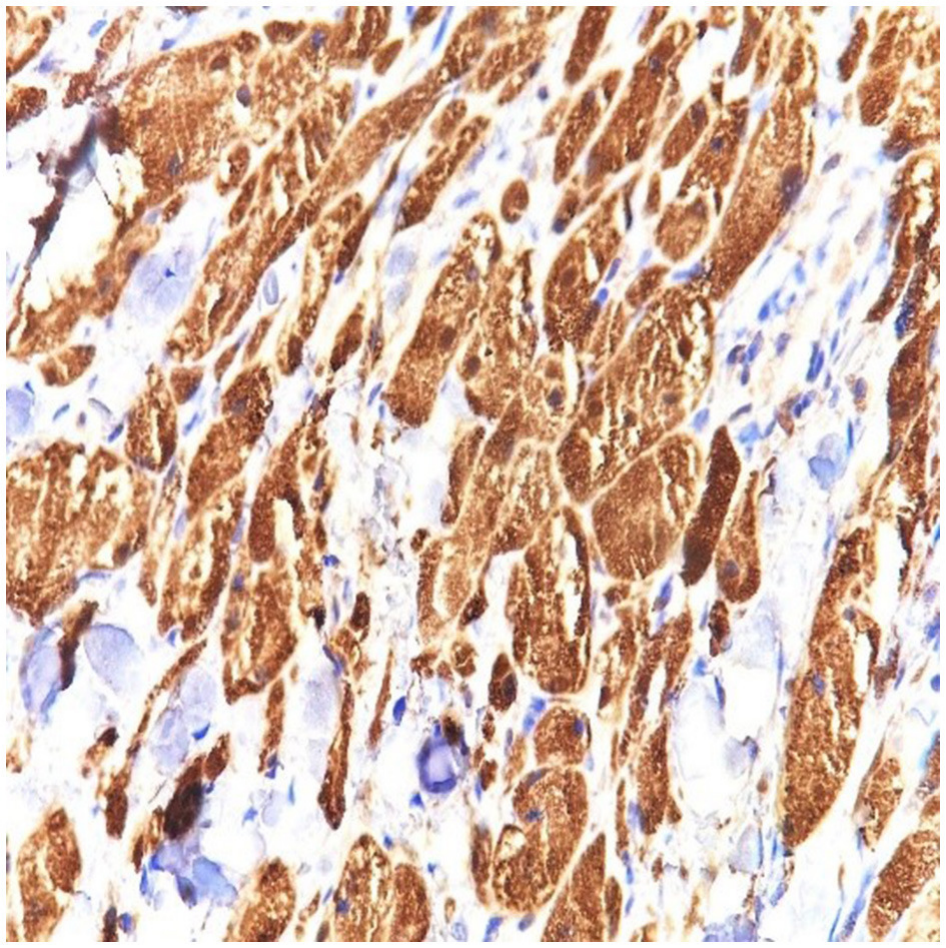
S100 stain in granular cell tumor strongly stains the cytoplasm and the nuclei of the granular cells. Strong and diffuse S100 expression is characteristic of this tumor (40 × magnification).

S100 protein was diffusely expressed by the entire tumor and was not expressed at the level of the resection limit, demonstrating the complete excision of the tumor mass, with the exception of an area of tumor cells extending around a nerve strand that reaches to the deeper margins of the resected lesion (Figures 2–4 correspond to the microscopic aspect of the tumor).

The cytoplasms of the tumor cells stain positively for CD68 in a granular manner.

Staining for tyrosinase was negative in the tumor but positive in the melanocytes, which were of normal number at the dermoepidermal junction. PRAME (Preferentially Expressed Antigen in Melanoma) was not expressed by tumor cells. The postoperative course was without complications.

## 3. Discussion

The etiology of these tumors is not known; initially, they were thought to be derived from myoblasts, and this fact is supported by the usual infiltration in the muscle fibers and by the damage of the tongue, a muscular organ (6). They were subsequently considered as developmental cells, histiocytes, neuroendocrine cells, fibroblasts, or undifferentiated mesenchymal cells (1). Fisher and Wechsler first showed by electron microscopy that these tumors are derived from nerve cells (7). In 1990, Mozur, Schultz, and Myers, using specific antibodies, also confirmed that GCT was derived from nerve cells (8), and with the advent of immunohistochemistry, it was established that GCT was derived from Schwann cells (9). GCTs are not common in children, they appear more between 30 and 50 years of age, and the distribution by sex is not clear in some studies predominates in women (9, 10), and in other studies, it predominates in men (11–13).

In terms of location, in order of frequency, they can be found at the level of the tongue (the most common) followed by the torso and limbs (5). Most of the time the tumor is solitary, but there can be multiple foci, including in the internal organs, with the percentage of multiple localizations reaching, according to some studies, up to 30% of cases (4). Multiple localizations in adults have been associated with some diseases such as neurofibromatosis and Hodgkin's lymphoma (4), and in children, they have been associated with cryptorchidism, pulmonary stenosis, congenital heart disease, or Noonan syndrome (14). Noonan syndrome is part of a group of diseases called *RASopathies*, characterized by genetic mutations in *RAS* proteins, that play an important role in cell differentiation and development, comprising Noonan syndrome and neurofibromatosis, Legius syndrome, and Costello syndrome and Leopard (PTPN1 gene mutation) (5, 15). Extracutaneous localization may affect the mammary gland, mediastinum, thyroid, larynx and trachea, lungs, ovary, testicle, heart, digestive tract, urinary tract, and rarely the central nervous system, but the central nervous system has the most severe clinical manifestations (1). At the breast level, GCT represents < 0.1% of all tumors and < 6% of all GCT tumors, and the preferred location being the superior-internal quadrant (16).

Clinically, it manifests as a round, painless, skin-colored tumor, located subcutaneously, with slow growth, with somewhat unclear margins, measuring between 5 mm and 10 cm in diameter, sometimes with a warty appearance due to epidermal hyperplasia (5, 6, 17). As the tumor affects the innervation of the skin, sometimes skin contractions occur. Malignancy is very rare, < 2% of cases, and is considered clinically as malignant only when they metastasize and when the size of the tumor exceeds 4 cm (18, 19). Malignant tumors have an accelerated growth rate and can cause metastasis to the lungs, bones, and brain, and metastases can be detected by positron emission tomography and F-18 fluorodeoxyglucose (16, 18). The differential diagnosis comprises lipoma, dermatofibroma, fibro-histiocytoma, pilar cyst, basal cell carcinoma, squamous cell carcinoma, pilomatrixoma, and other types of lesions (20). When they appear on the breast, they may look like breast cancer, but the therapeutic behavior and prognosis are completely different, and sometimes they can be concomitant with invasive ductal carcinoma either in the same breast or in the opposite breast (14, 21).

What is important to follow is the potential conversion from benign to malignant, which is a necessary clinical and histopathological correlation because some very precise criteria of malignancy are missing only on a histological basis. Other diagnostic methods, which may permit a more accurate assessment of the degree of malignancy as well as prognosis in such cases may exist (22).

Histologically, GCT is an unencapsulated tumor consisting of large polyhedral cells with small hyperchromatic central nuclei and a cytoplasm with abundant eosinophilic granules due to the accumulation of secondary lysosomes in the cytoplasm, often extending to the superficial hypodermis. Tumor cells often look like large eosinophilic granules surrounded by a transparent halo known as Milian's pustulo-ovoid bodies, the number of which increases with the age of the tumor (11). Occasionally, binuclear cells, stripped nuclei, dirty nuclei, and intranuclear inclusions may also be observed. The overlying epithelium is often characterized by prominent pseudoepitheliomatous hyperplasia that can be confused with squamous cell carcinoma if the biopsy is taken superficially. Pseudoepitheliomatous hyperplasia can be considered not as a tumor extension but rather as a reaction-type change in the underlying tumor (21).

Immunohistochemistry is positive for S100 protein, CD68 antigen (KP-1), and (neuron-specific enolase) (NSE). Some tumors may be S100-negative and are known as non-neural GCTs; these tumors have recently been reported to overexpress ALK and cyclin D1 and are probably different entities (22).

Fanburg and Smith proposed a classification to evaluate the malignancy of these tumors. Thus, he proposed the following parameters of analysis (23):

- Necrosis
- Spindling
- Vesicular nuclei with large nucleoli
- >2 mitoses/10 high-power fields at × 200 magnification
- High nuclear-to-cytoplasmic ratio
- Pleomorphism

Tumors that did not meet any of these criteria were considered benign, and those that met one or two criteria were considered atypical, and those with three or more were classified as malignant.

Since we are discussing about a pediatric case, the first differential diagnosis would be a spitzoid melanocytic tumor; therefore, tyrosinase and PRAME were performed. In our case, we used tyrosinase, which is positive in melanocytic proliferations and negative in Abrikossoff tumor.

From a histopathologic point of view, we can consider also other differential diagnoses, such as congenital granular cell epulis, cutaneous non-neural granular cell tumor, hibernoma, malignant melanoma, and granular cell dermatofibroma.

Other innovative imaging techniques can be used for diagnosis, such as optical coherence tomography (OCT). Optical coherence tomography (OCT) is an emerging imaging technique that is capable of acquiring high-resolution cross-sectional images of a tissue, being similar to ultrasound; however, infrared waves are used instead of sound waves (24).

The OCT images of the GCT reveal verrucous epidermal hyperplasia, seen as hyperreflective, uneven surface of the tissue. The dermo-epidermal junction is obscured in the OCT images of GCT, while it is discernible in the adjacent healthy skin. Blood vessels are visible in the dermis of the healthy skin but not in the images of GCT (25). Other emerging imaging techniques, such as reflective confocal microscopy, photoacoustic imaging, may also be combined with OCT to improve the diagnosis of GCT (26).

The simple excision of the tumor is the treatment of choice, followed by histopathological examination and possibly immunohistochemical examination. Only if the resection edges are positive is secondary recovery recommended. In malignant tumors, surgical treatment can be supplemented with chemotherapy or radiation therapy, the benefits of which remain unclear (27). The recommended monitoring is done for 10 years as the data from the literature show a local recurrence rate of up to 8% for situations with surgical negative margins and nearly 20% for those with positive surgical margins (28).

Abrikossoff tumor is a rare entity; thus, our case will contribute to growing the body of evidence on its presentation and potential therapy, as well as pave the way for further research. We did not report possible correlations with the COVID “era” nor did we find associations with SARS-CoV 2 infection, as in other cases (29).

## 4. Conclusion

GCT are a rare entity. They are usually benign, occasionally malignant. The diagnosis is based on the S100 immunohistochemical examination. The treatment of choice was simple surgical excision. In the case of benign tumors, the evolution and prognosis are favorable. Clinicians should be aware of its existence and bear it in mind as a possible differential diagnosis.

## Data availability statement

The original contributions presented in the study are included in the article/supplementary material, further inquiries can be directed to the corresponding authors.

## Ethics statement

The study was conducted in accordance with the Declaration of Helsinki and it was conducted in a private clinic of plastic and aesthetics surgery procedures. It was approved by the Institutional Review Board of the Arestetic Clinic from Galaţi, Romania (63/04.07.2022). Written informed consent to participate in this study was provided by the participants' legal guardian/next of kin. Written informed consent was obtained from the individual's next of kin for the publication of any potentially identifiable images or data included in this article.

## Author contributions

VA, RJ, and AT: conceptualization. TT, FB, and RT: software. VA, FB, and AT: validation. VA: methodology, formal analysis, data curation, and writing—original draft preparation. VA, LM, and MM: resources. LN, LM, and MM: writing—review and editing. FB, LM, and MM: visualization. TT: supervision. All authors have read and agreed to the final version of the manuscript.
